# Skeletal muscle quality as assessed by CT-derived skeletal muscle density is associated with 6-month mortality in mechanically ventilated critically ill patients

**DOI:** 10.1186/s13054-016-1563-3

**Published:** 2016-12-01

**Authors:** Wilhelmus G. P. M. Looijaard, Ingeborg M. Dekker, Sandra N. Stapel, Armand R. J. Girbes, Jos W. R. Twisk, Heleen M. Oudemans-van Straaten, Peter J. M. Weijs

**Affiliations:** 1 Department of Intensive Care Medicine, VU University Medical Center Amsterdam, De Boelelaan 1117, Amsterdam, The Netherlands; 2 Institute for Cardiovascular Research, VU University Medical Center Amsterdam, De Boelelaan 1117, Amsterdam, The Netherlands; 3 Department of Nutrition and Dietetics, Internal Medicine, VU University Medical Center Amsterdam, De Boelelaan 1117, 1081 HV Amsterdam, The Netherlands; 4 Department of Epidemiology and Biostatistics, VU University Medical Center Amsterdam, van der Boechorststraat 7, Amsterdam, The Netherlands; 5 Department of Nutrition and Dietetics, Amsterdam University of Applied Sciences, Dr. Meurerlaan 8, Amsterdam, The Netherlands; 6 VU University Medical Center Amsterdam, Room ZH 7D174, P.O. Box 7057, 1007 MB Amsterdam, The Netherlands

**Keywords:** Intensive care unit, Computed tomography, CT, Muscle, Muscle quality, Myosteatosis, Skeletal muscle density, Intermuscular adipose tissue, Mortality, Outcome

## Abstract

**Background:**

Muscle quantity at intensive care unit (ICU) admission has been independently associated with mortality. In addition to quantity, muscle quality may be important for survival. Muscle quality is influenced by fatty infiltration or myosteatosis, which can be assessed on computed tomography (CT) scans by analysing skeletal muscle density (SMD) and the amount of intermuscular adipose tissue (IMAT). We investigated whether CT-derived low skeletal muscle quality at ICU admission is independently associated with 6-month mortality and other clinical outcomes.

**Methods:**

This retrospective study included 491 mechanically ventilated critically ill adult patients with a CT scan of the abdomen made 1 day before to 4 days after ICU admission. Cox regression analysis was used to determine the association between SMD or IMAT and 6-month mortality, with adjustments for Acute Physiological, Age, and Chronic Health Evaluation (APACHE) II score, body mass index (BMI), and skeletal muscle area. Logistic and linear regression analyses were used for other clinical outcomes.

**Results:**

Mean APACHE II score was 24 ± 8 and 6-month mortality was 35.6%. Non-survivors had a lower SMD (25.1 vs. 31.4 Hounsfield Units (HU); *p* < 0.001), and more IMAT (17.1 vs. 13.3 cm^2^; *p* = 0.004). Higher SMD was associated with a lower 6-month mortality (hazard ratio (HR) per 10 HU, 0.640; 95% confidence interval (CI), 0.552–0.742; *p* < 0.001), and also after correction for APACHE II score, BMI, and skeletal muscle area (HR, 0.774; 95% CI, 0.643–0.931; *p* = 0.006). Higher IMAT was not significantly associated with higher 6-month mortality after adjustment for confounders. A 10 HU increase in SMD was associated with a 14% shorter hospital length of stay.

**Conclusions:**

Low skeletal muscle quality at ICU admission, as assessed by CT-derived skeletal muscle density, is independently associated with higher 6-month mortality in mechanically ventilated patients. Thus, muscle quality as well as muscle quantity are prognostic factors in the ICU.

**Trial registration:**

Retrospectively registered (initial release on 06/23/2016) at ClinicalTrials.gov: NCT02817646.

## Background

Muscle wasting is a severe complication of critical illness [1]. Puthucheary et al. reported a steady decrease in skeletal muscle mass of almost 20% during the first 10 days of intensive care unit (ICU) admission [2]. Loss of muscle has been associated with longer duration of mechanical ventilation and higher ICU and hospital mortality [3–5]. If patients survive, they exhibit long-term functional disability with a great impact on quality of life for as long as 5 to 8 years after admission [6–8]. However, many patients already have a low muscle quantity upon admission to the ICU. In two retrospective studies as much as 60–70% of patients had low muscle quantity as assessed on computed tomography (CT) scans on ICU admission, and low muscle quantity at ICU admission was associated with a higher mortality [9, 10].

Not only the quantity, but also the quality of muscle seems important [11]. Along with a decline in muscle mass, fatty infiltration of muscles or myosteatosis has been identified as a possible cause of loss of muscle quality [11]. Myosteatosis can be apparent within muscle fibres and evaluated on CT scans by measuring skeletal muscle density (SMD), or between muscle fibres and evaluated on CT scans by measuring the amount of adipose tissue between muscles (also termed intermuscular adipose tissue or IMAT). A lower SMD was associated with increased lipid infiltration in muscle biopsies and poor clinical outcomes in non-ICU populations [12–14]. Additionally, a recent study in critically ill patients using ultrasound of the quadriceps muscle found that not only a decrease in muscle quantity but also increased muscle echogenicity was related to a decrease in muscle function [15]. An increased amount of IMAT as assessed on CT scans has been associated with decreased muscle function and increased (systemic) inflammation in non-ICU populations [16, 17]. The aim of the present study was to investigate if muscle quality, as assessed by CT-derived SMD and IMAT, is associated with mortality independently of muscle quantity and severity of illness. We hypothesized that low SMD and high IMAT at ICU admission are associated with a poor outcome, independent of the quantity of muscle and severity of illness.

## Methods

### Patients and data

This is a retrospective analysis of CT-derived muscle quality at a single time point at ICU admission in critically ill patients admitted to a m﻿﻿ixed medical-surgical ICU of a university hospital from September 2003 to April 2013. Patients were included if they were aged 18 years or older, stayed in the ICU for at least 4 days, required mechanical ventilation during their ICU stay, and had an abdominal CT scan made 1 day before or up to 4 days after admission to the ICU. Patients were excluded if the CT scan was not eligible for analysis, or if data on body weight or height or the Acute Physiological, Age, and Chronic Health Evaluation (APACHE) II score was missing. By searching the hospital information system for any patients meeting inclusion criteria, we expanded our previously reported cohort of ICU patients [9].

Patient data including age, sex, weight, height, admission diagnosis, APACHE II score, length of ventilation (LOV), ICU length of stay (ICU-LOS) and hospital length of stay (hospital-LOS), discharge destination, and ICU and hospital mortality was obtained from the ICU patient data management system (Metavision; IMDsoft, Tel-Aviv, Israel) and the hospital information system (Mirador; iSOFT Nederland BV, Leiden, The Netherlands). If mortality data were not registered, these were collected from the civil registry or from the general practitioner.

### CT scan analysis

The precision of single slice CT scan analysis at the third lumbar vertebra (L3) level is high (inter- and intra-observer variability less than 2% in healthy volunteers) [18]. Both skeletal muscle area (*r* = 0.83–0.99; *p* < 0.01) and IMAT (*r* = 0.39–0.61; *p* < 0.05) at this level are closely related to whole body skeletal muscle and IMAT volumes as assessed by magnetic resonance imaging (MRI) [19–21].

CT scans made 1 day before to 4 days after ICU admission for diagnostic purposes were imported from the hospital radiology system and stored on a secure computer system. Scans were analysed using Slice-O-matic versions 4.3 and 5.0 (TomoVision, Montreal, QC, Canada) by two trained and certified investigators (WGPML and IMD, trained by the Cross Cancer Institute, Edmonton, AB, Canada) who had frequent consultation with each other if there was any doubt about eligibility, landmarking, or analysis.

The CT scans were analysed for eligibility and rejected if the scan quality was too low for analysis or if they contained artefacts, or if muscle was cut off due to windowing. Landmarking was performed by identifying the L3 and isolating the CT slice that depicted the whole vertebra the best. A bony landmark was used to ensure reproducibility and consistency between patients.

**Fig. 1:**
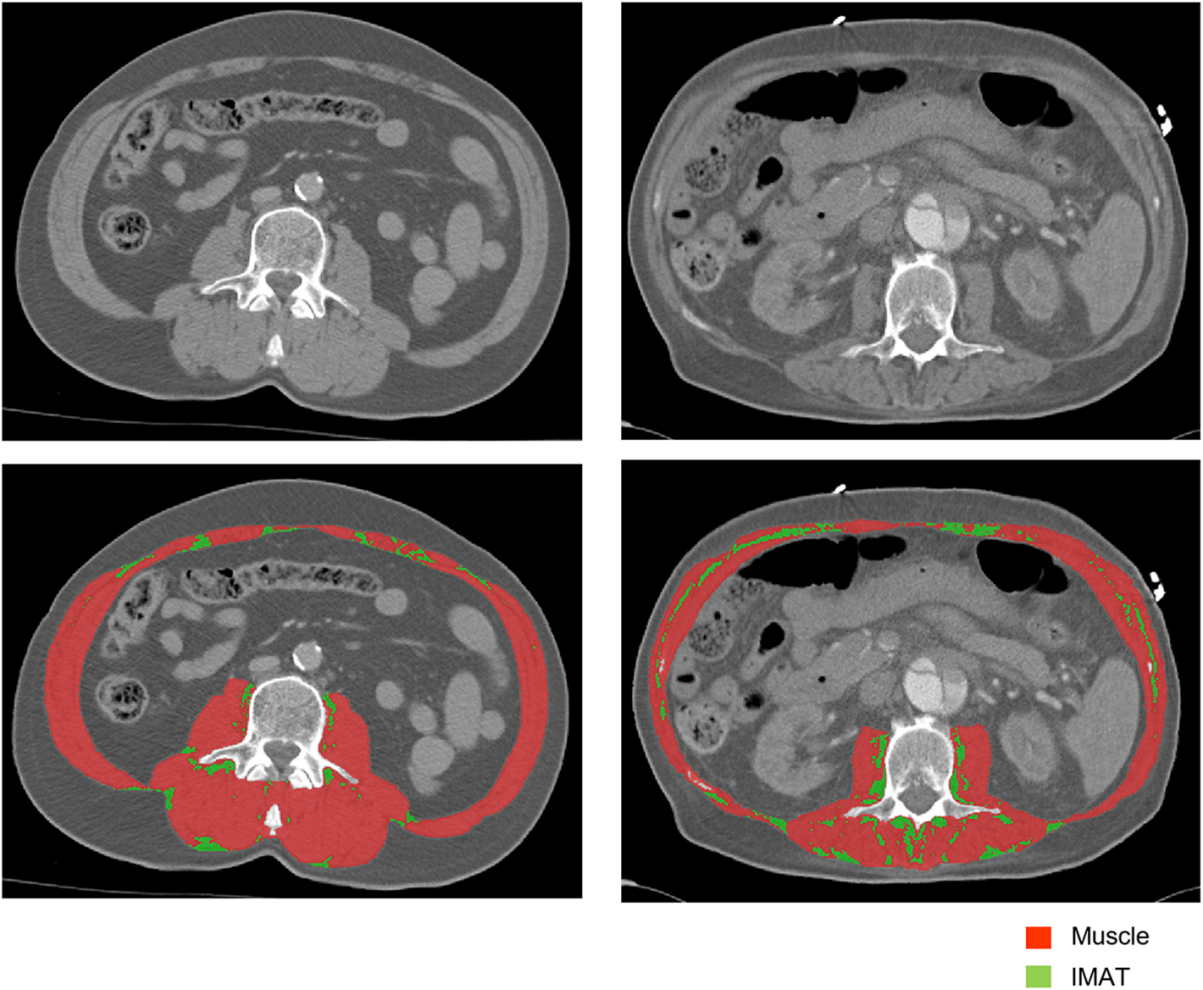
Example of CT scan analysis. This image shows CT scans at the level of lumbar vertebra 3 of two patients both un-analysed (*upper row*) and analysed (*lower row*). The analysed images show muscle tissue (*red*) and intermuscular adipose tissue (*IMAT*, *green*). The patient on the left has more muscle (165 vs. 120 cm^2^), less IMAT (10 vs. 19.5 cm^2^), and higher mean skeletal muscle density (42 vs. 18 Hounsfield Units) than the patient on the right

Different tissues were identified using boundaries in Hounsfield Units (HU) set to –29 to +150 for muscle, –190 to –30 for IMAT and subcutaneous adipose tissue, and –150 to –50 for visceral adipose tissue [22]. SMD was assessed by the mean radiological muscle attenuation of all muscle visible at the L3 level, measured in HU. The HU scale is a radiological scale describing the density of tissues on CT scans [23]. Lower mean muscle attenuation indicates less dense muscle tissue with more lipid infiltration, e.g. lower SMD, while a higher mean muscle attenuation indicates denser muscle tissue with less lipid infiltration, e.g. higher SMD [14]. IMAT was assessed by identifying all visible adipose tissue within muscle fascia in cm^2^ [22]. Previously found ICU-specific optimal cut-off points related to hospital mortality were used to define low skeletal muscle area: below 170 cm^2^ for male patients and below 110 cm^2^ for female patients [9]. See Fig. 1 for an example of CT scan analysis.

Because muscle quality is important for dealing with recovery after ICU and hospital discharge, we chose 6-month mortality as the primary endpoint. Secondary endpoints were the odds of being discharged from the hospital to home, length of ventilation, and ICU and hospital LOS in survivors.

### Statistics

Independent sample *t* tests were used to compare survivors and non-survivors for normally distributed continuous variables, and Mann-Whitney *U* tests for non-normally distributed continuous variables. Fisher exact and Chi^2^ tests with post-hoc Bonferroni analysis were used to compare survivors and non-survivors for categorical variables. Kaplan-Meier plots were made to visualize the effect of SMD and IMAT (divided into two groups based on the median) on 6-month mortality, with log-rank tests to compare the survival curves of the two groups. Cox regression analysis was used to evaluate the association between SMD or IMAT (as continuous variables) and 6-month mortality. After univariable analyses, APACHE II score was added to the models to adjust for severity of illness (model 2). In the second adjusted model, body mass index (BMI), and skeletal muscle area were included as well (model 3). Age is included in the APACHE II score and was therefore not separately included in the adjusted models. Additionally, we performed analyses on the subgroup of patients with available data on visceral and subcutaneous adipose tissue in which BMI was substituted with visceral and subcutaneous adipose tissue as a measure of total body fatness (model 4).

Logistic and linear regression analyses were used to evaluate the association between SMD or IMAT and the secondary outcome measures discharge to home, LOV, ICU-LOS, and hospital-LOS in survivors. LOV, ICU-LOS, and hospital-LOS were non-normally distributed and positively skewed; therefore, the analysis was performed on the natural logarithm of the variables. By re-transforming by using the inverse, the influence of a given predictor was calculated as a percentage change in outcome.

IBM SPSS Statistics 22 (IBM Corp, Armonk, NY, USA) was used for statistical analysis. Values are reported as mean ± standard deviation (SD) or median and 25–75% interquartile range (IQR). All statistical tests were two-sided. A *p* < 0.05 was considered statistically significant.

## Results

**Fig. 2:**
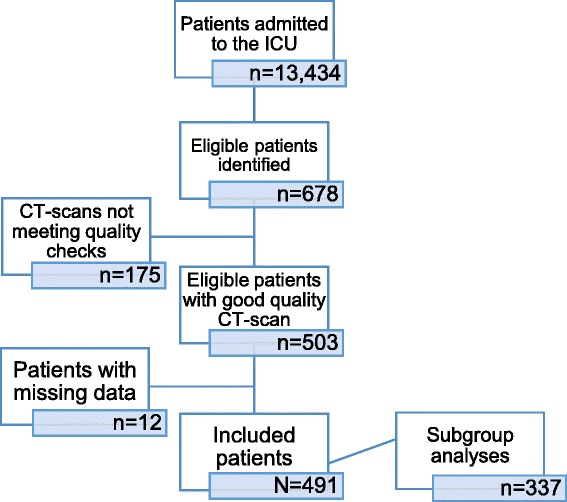
Consort diagram showing the inclusion process. *CT* computed tomography, *ICU* intensive care unit

A total of 13,434 patients were admitted to the ICU during the study period with a mean APACHE II score of 17.4 ± 9.2. Six hundred and seventy-eight patients fulfilled inclusion criteria and had their CT scans imported from the radiology system to be analysed for eligibility. CT scans that were found not to be eligible were due to artefacts (78 scans), muscle cut-off (50 scans), or low quality (47 scans). Finally, 491 patients (72%) with complete clinical data and good quality CT scans were included for the statistical analysis. However, due to windowing or artefacts, visceral and/or subcutaneous adipose tissue could not be analysed in 154 patients. We therefore performed subgroup analyses that included visceral and subcutaneous adipose tissue in a subgroup of 337 patients (50%). Figure 2 is the consort diagram showing the inclusion process.

### Patient characteristics

Patient characteristics are presented in Table 1 for 6-month survivors and non-survivors. Outcome measures are presented separately in Table 2. CT scans were mostly made on the day of admission to the ICU. Three hundred and twelve (64.7%) patients had a low skeletal muscle area at ICU admission. Six-month mortality was 35.6%. Non-survivors were older (67 ± 14 vs. 55 ± 18 years; *p* < 0.001), had a lower BMI (24.6 ± 4.3 vs. 25.5 ± 4.4 kg/m^2^; *p* = 0.042), higher APACHE II score (27 ± 8 vs. 22 ± 8; *p* < 0.001), and were more often medical patients (62% vs. 43%; *p* < 0.001) than survivors.

**Table 1:** Patient characteristics of all patients and comparison between survivors and non-survivors

	All patients *N* = 491		Survivors^1^ (*n* = 299)		Non-survivors^1^ (*n* = 165)		*P* value survivors vs. non-survivors
	Mean/median/*n*	SD/IQR/%	Mean/median/*n*	SD/IQR/%	Mean/median/*n*	SD/IQR/%	
Age, years	58	±18	55	±18	67	±14	**<0.001**
Sex, male, *n* (%)	305	62%	191	64%	93	56%	0.135
BMI, kg/m^2^	25.2	±4.3	25.5	±4.4	24.6	±4.3	**0.042**
Underweight^2^, *n* (%)	19	4.1%	11	3.7%	8	4.8%	0.291
Normal weight^2^, *n* (%)	238	51.3%	145	48.5%	93	56.4%	
Overweight^2^, *n* (%)	158	34.1%	108	36.1%	50	30.3%	
Obesity^2^, *n* (%)	49	10.6%	35	11.7%	14	8.5%	
APACHE II score	24	±8	22	±8	27	±8	**<0.001**
Admission category, *n* (%)							**0.001**
Medical	248	50.5%	130	43%	102	62%	
Surgical	243	49.5%	169	57%	63	38%	
Admission diagnosis, *n* (%)							**<0.001**
Cardiovascular	32	6.5%	18^a^	6.0%	14^a^	8.5%	
Metabolic/renal	15	3.1%	8^a^	2.7%	6^a^	3.6%	
Neurologic	41	8.4%	19^a^	6.4%	16^a^	9.7%	
Post-resuscitation	28	5.7%	16^a^	5.4%	11^a^	6.7%	
Post-surgery	149	30.3%	95^a^	31.8%	50^a^	30.3%	
Respiratory insufficiency	68	13.8%	40^a^	13.4%	25^a^	15.2%	
Sepsis	31	6.3%	14^a^	4.7%	15^a^	9.1%	
Trauma	94	19.1%	74^a^	24.7%	13^b^	7.9%	
Other	33	6.7%	15^a^	5.0%	15^a^	9.1%	
Length of hospital stay before ICU admission, days	0	0–4	0	0–4	0	0–6	0.166
Time from ICU admission to CT scan, days	0	0–1	0	0–1	0	0–1	0.277
Skeletal muscle area, cm^2^	136.5	±39.0	143.5	±38.9	120.3	±33.0	**<0.001**
Skeletal muscle index, cm^2^/m^2^	44.8	±11.0	46.6	±10.6	40.4	±9.9	**<0.001**
Low skeletal muscle area^3^, *n* (%)	312	63.5%	163	54.5%	137	83.0%	**<0.001**
SMD, HU	29.9	±11.7	31.4	±11.7	25.1	±9.4	**<0.001**
IMAT, cm^2^	13.6	8.4–24.3	13.3	7.9–23.2	17.1	10.5–27.1	**0.004**
Visceral adipose tissue, cm^2^ (*n* = 337)	96.7	49.3–170.6	95.8	50.9–178.1	108.1	54.1–177.5	0.593
Subcutaneous adipose tissue, cm^2^ (*n* = 337)	132.7	90.2–182.4	133.7	89.8–189.2	127.7	95.7–176.2	0.440

^1^Survivors and non-survivors 6 months after ICU admission

^2^WHO categories: underweight, BMI <18.5; normal weight: BMI 18.5–24.9; overweight: BMI 25–29.9; obesity: BMI ≥30 [42]

^3^Defined by skeletal muscle area: <170 cm^2^ for males and <110 cm^2^ for females [9]

^a, b^Values in the same row not sharing the same superscript letter are significantly different in a post-hoc Bonferroni analysis

Values in bold indicate statistically significant *p* values

*APACHE* Acute Physiological, Age, and Chronic Health Evaluation, *BMI*, body mass index, *CT* computed tomography, *HU* Hounsfield Units, *ICU* intensive care unit, *IMAT* intermuscular adipose tissue, *IQR* interquartile range, *SD* standard deviation, *SMD* skeletal muscle density

**Table 2:** Primary and secondary outcome measures

	*n*	%	Days	IQR
Six-month mortality	165	35.6%		
ICU mortality	84	17.1%		
Hospital mortality	132	26.9%		
Length of ventilation			11	6–20
ICU length of stay			13	7–23
Hospital length of stay			35	19–59
Destination after discharge				
Home	144	40.7%		
Other hospital	80	22.6%		
Nursing home	76	21.5%		
Rehabilitation unit	45	12.7%		
Other	9	2.5%		

*ICU* intensive care unit, *IQR* interquartile range

Mean SMD at ICU admission was 29.9 ± 11.7 HU. Median IMAT at ICU admission was 13.6 (8.4–24.3) cm^2^, comprising 9.1% of total tissue within muscle fascia (skeletal muscle area plus IMAT) at the L3 level. Non-survivors had a lower skeletal muscle area (120.3 ± 33.0 vs. 143.5 ± 38.9 cm^2^; *p* < 0.001), lower SMD (25.1 ± 9.4 vs. 31.4 ± 11.7 HU; *p* < 0.001), and more IMAT (17.1 (10.5–27.1) vs. 13.3 (7.9–23.2) cm^2^; *p* = 0.004) than survivors.

### Association between muscle quality and 6-month mortality

**Fig. 3:**
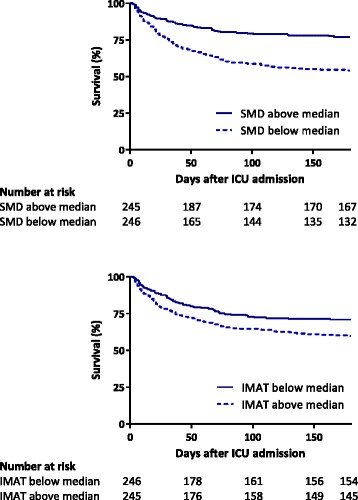
Kaplan-Meier plots. These graphs illustrate mortality for groups below and above median skeletal muscle density (*SMD*) (29.2 Hounsfield Units) and median intermuscular adipose tissue (*IMAT*) (13.6 cm^2^). *ICU* intensive care unit

Mortality was significantly higher in patients with low muscle quality with SMD values below the median or IMAT values above the median (Fig. 3).

Cox regression analysis showed that higher SMD was associated with lower 6-month mortality (hazard ratio (HR) per 10 HU, 0.640; 95% confidence interval (CI), 0.552–0.742; *p* < 0.001; Table 3). This association was still apparent when SMD was adjusted for the confounders APACHE II score, BMI, and skeletal muscle area (HR per 10 HU, 0.774; 95% CI, 0.643–0.931; *p* = 0.006).

**Table 3:** Cox regression: association between skeletal muscle density or intermuscular adipose tissue and mortality

	Univariable *N* = 491			Model 2 *N* = 491			Model 3 *N* = 491			Model 4 (*n* = 337)		
6-month mortality	HR	95% CI	*P* value	HR	95% CI	*P* value	HR	95% CI	*P* value	HR	95% CI	*P* value
SMD (per 10 HU)	0.640	0.552–0.742	**<0.001**	0.703	0.605–0.818	**<0.001**	0.774	0.643–0.931	**0.006**	0.728	0.571–0.928	**0.010**
IMAT (per 10 cm^2^)	1.153	1.042–1.277	**0.006**	1.092	0.980–1.217	0.110	1.092	0.966–1.236	0.159	1.244	1.048–1.476	**0.012**

Model 2: adjusted for APACHE II score

Model 3: adjusted for APACHE II score, skeletal muscle area, and BMI

Model 4 (subgroup analysis): adjusted for APACHE II score, skeletal muscle area, visceral adipose tissue, and subcutaneous adipose tissue

Values in bold indicate statistically significant *p* values

*APACHE* Acute Physiological, Age, and Chronic Health Evaluation, *CI* confidence interval, *HR* hazard ratio, *HU* Hounsfield Units, *IMAT* intermuscular adipose tissue, *SMD* skeletal muscle density

Cox regression analysis showed that higher IMAT was associated with higher 6-month mortality (HR per 10 cm^2^, 1.153; 95% CI, 1.042–1.277; *p* = 0.006). However, when adjusted for APACHE II score alone or the confounders APACHE II score, BMI, and skeletal muscle area the association between IMAT and 6-month mortality was not significant (HR per 10 cm^2^, 1.092; 95% CI, 0.966–1.236; *p* = 0.159).

### Analyses in the subgroup with visceral and subcutaneous adipose tissue

Additional Cox regression analyses were performed in the subgroup of patients with available data on visceral and subcutaneous adipose tissue (*n* = 337, Table 3). Patients in this subgroup were significantly different from patients in whom visceral and/or subcutaneous adipose tissue could not be analysed. They were younger (56 vs. 64 years; *p* < 0.001), more often male (66 vs. 55%; *p* = 0.021), and had a lower BMI (24.8 vs. 25.9 kg/m^2^; *p* = 0.026). In this subgroup, we found both SMD (HR per 10 HU, 0.623; 95% CI 0.524–0.739; *p* < 0.001) and IMAT (HR per 10 cm^2^, 1.245; 95% CI, 1.106–1.401; *p* < 0.001) were significantly associated with 6-month mortality. In multivariable analyses both SMD (HR per 10 HU, 0.728; 95% CI, 0.571–0.928; *p* = 0.010) and IMAT (HR per 10 cm^2^, 1.244; 95% CI, 1.048–1.476; *p* = 0.012) remained significantly associated with 6-month mortality, adjusted for APACHE II score, skeletal muscle area, and visceral and subcutaneous adipose tissue.

### Secondary outcome measures in survivors

Higher SMD was significantly associated with shorter hospital-LOS after adjustment for APACHE II score, BMI, and skeletal muscle area (Table 4). After re-transformation we found that 10 HU higher SMD was associated with a 14% shorter hospital-LOS. IMAT was not associated with hospital LOS. Neither SMD nor IMAT were significantly associated with the odds of being discharged to home, LOV, or ICU-LOS.

**Table 4:** Logistic and linear regression: association between skeletal muscle density or intermuscular adipose tissue and secondary outcomes

	Univariable			Model 2			Model 3		
	OR/B	95% CI	*P* value	OR/B	95% CI	*P* value	OR/B	95% CI	*P* value
Discharge to home									
SMD (per 10 HU)	1.039	0.864 to 1.250	0.683	0.990	0.816 to 1.200	0.915	0.926	0.718 to 1.195	0.556
IMAT (per 10 cm^2^)	0.886	0.741 to 1.059	0.182	0.912	0.761 to 1.093	0.317	0.884	0.715 to 1.093	0.254
Length of ventilation									
SMD (per 10 HU)	–0.038	–0.107 to 0.032	0.292	–0.003	–0.075 to 0.069	0.936	–0.018	–0.111 to 0.075	0.705
IMAT (per 10 cm^2^)	0.050	–0.014 to 0.115	0.126	0.029	–0.036 to 0.094	0.384	0.026	–0.049 to 0.101	0.499
Length of ICU stay									
SMD (per 10 HU)	–0.051	–0.123 to 0.020	0.158	–0.020	–0.092 to 0.053	0.598	–0.032	–0.128 to 0.063	0.506
IMAT (per 10 cm^2^)	0.064	–0.003 to 0.130	0.059	0.043	–0.023 to 0.110	0.199	0.041	–0.036 to 0.119	0.292
Length of hospital stay									
SMD (per 10 HU)	–0.123	–0.192 to –0.054	**0.001**	–0.112	–0.184 to –0.041	**0.002**	–0.134	–0.228 to –0.040	**0.005**
IMAT (per 10 cm^2^)	0.075	0.010 to 0.140	**0.023**	0.065	–0.001 to 0.131	0.052	0.064	–0.012 to 0.141	0.100

Model 2: adjusted for APACHE II score

Model 3: adjusted for APACHE II score, skeletal muscle area, and BMI

Discharge to home results are given as OR; length of ventilation, ICU, and hospital stay are given as B values

Values in bold indicate statistically significant *p* values

*APACHE* Acute Physiological, Age, and Chronic Health Evaluation, *B* beta coefficient, *CI* confidence interval, *HU* Hounsfield Units, *ICU* intensive care unit, *IMAT* intermuscular adipose tissue, *OR* odds ratio, *SMD* skeletal muscle density

## Discussion

This retrospective study in mechanically ventilated patients admitted to the ICU for 4 days or longer shows that low skeletal muscle quality at ICU admission, as assessed by skeletal muscle density on CT scans, is associated with higher 6-month mortality independent of muscle quantity, APACHE II score, and BMI. A lower SMD was also associated with a longer hospital stay in survivors. This is the first study investigating the relation between CT-derived markers for muscle quality and outcome in ventilated critically ill patients. Intermuscular adipose tissue was also associated with mortality but not independently, suggesting that SMD is a stronger marker of muscle quality for 6-month mortality or that IMAT is better represented by confounders than SMD.

### Muscle quality and quantity

Previously we have found that low muscle quantity as assessed by skeletal muscle area on CT scans at ICU admission is a risk factor for hospital mortality, independent of sex and APACHE II score [9]. These findings were in line with a study by Moisey et al. in elderly injured ICU patients, who found low skeletal muscle area to be associated with higher mortality and less ventilator-free and ICU-free days [10]. In the present study, we found that the quality of muscle appeared to be important for survival in addition to quantity.

The APACHE II score is the best validated prognostic ICU score for hospital mortality incorporating age, comorbidities, and acute illness. However, it appears that, independently of APACHE II score, a poor health status as reflected by low muscle quantity and quality (whether due to inactivity, comorbidity, or high age) are important prognostic markers. Unfortunately, the updated APACHE III and IV scores were not available for all patients.

Of interest, IMAT was independently associated with 6-month mortality in a subgroup, but not in the entire cohort. The patients in the subgroup were younger, more often male, and had a lower BMI. Apparently, visceral tissue on CT scans can more often not be analysed in older patients with high BMI, mostly because a part of the scan is often cut-off in the windowing process.

### Causes and consequences of myosteatosis

Previous studies have shown that inactivity, as seen in pre-existing illness and advancing age, can cause an increase in myosteatosis (as seen by a decrease in SMD and an increase in IMAT) and that these changes are associated with decreased muscle strength [24–26]. During inactivity there is a decrease in lipoprotein lipase activity, the rate-limiting enzyme in triglyceride metabolism, which hydrolyses triglycerides into lipoproteins [27, 28]. Additionally, during bed rest a decrease in 3-hydroxyacyl-CoA-dehydrogenase concentration is seen, which impairs the muscle’s ability to metabolize free fatty acids to acyl-CoA [29, 30]. Finally, denervation causes an increase in malonyl-CoA concentrations, which in turn inhibits the rate-limiting enzyme responsible for transporting acyl-CoA into the mitochondria [31]. These altered metabolic mechanisms associated with inactivity decrease the ability of muscles to oxidise lipids and promotes a shift in muscle fuel utilisation from lipids towards glucose, causing accumulation of lipids in the muscle [26, 32]. Manini et al. found that 4 weeks of lower limb immobilisation in healthy adults caused an increase in IMAT and a loss in muscle strength independent of a decrease in muscle mass [26]. Their findings support the idea that myosteatosis is related to decreased muscle quality.

Adipose tissue has been noted as a major endocrine organ. To date, hundreds of adipokines, cytokines secreted by adipose tissue, have been identified [33]. Myosteatosis is associated with an upregulation of macrophage and T-cell expression [34]. These inflammatory cells produce pro-inflammatory cytokines such as tumour necrosis factor-alpha (TNFα) and interleukin-6 (IL-6) [35] which mediate contractile dysfunction [36, 37] and create a low-grade inflammatory environment in which the metabolic syndrome, cardiovascular disease, and insulin resistance are prone to develop [16, 17, 34].

### Muscle wasting and long-term outcome

Previous studies have shown that muscle wasting as occurring *during* critical illness has a large impact on survival, successful weaning from ventilation, and long-term functioning [3–8, 38]. Herridge et al. found functional disability in survivors of acute respiratory distress syndrome as much as 5 years after admission to the ICU [7] and Iwashyna et al. found functional limitations up to 8 years after severe sepsis [8]. A decrease in muscle quality as assessed by CT scans has been described in 15 patients in a small substudy of the EPaNIC trial where a substantial decrease in skeletal muscle area and SMD, and an increase in IMAT developing over a 7-day period during the early stage of critical illness was found [39]. In two observational studies including 136 and 115 patients requiring at least 5 and 7 days of mechanical ventilation, respectively, muscle weakness acquired during critical illness was associated with increased ICU and hospital mortality [3, 38]. Our study found that low muscle quality present at the beginning of critical illness was already associated with poor outcome, before the devastating effects of critical illness on muscle wasting.

### Strengths and limitations

Our study has strengths and limitations. This is the first study up to now investigating the relation between muscle quality assessed with CT scans and clinical outcomes in a large group of critically ill ventilated patients. However, we only included patients who had a CT scan made and the resulting selection bias might limit the generalizability of our findings to the overall ICU population. The APACHE II score of the study population was higher than the overall ICU population, all patients were ventilated, and had an ICU length of stay of at least 4 days, indicating that the study patients were severely ill. Low muscle quality at admission likely has greater impact in the more severely ill patients, because the effect of additional critical illness-related muscle wasting is greater in this population.

Muscle quality is typically defined as muscle strength per unit of muscle mass or cross-sectional area. However, measuring muscle strength in ventilated critically ill patients is not feasible. Therefore, we used SMD and IMAT as proxy markers for muscle quality [40]. To date, SMD on CT scans has been related to myosteatosis [14, 23]. However, in recent studies in ICU patients using ultrasound, a relation between ultrasound echogenicity and myonecrosis in muscle biopsies has been found [2, 41]. Changes in SMD on CT scans might therefore not only reflect myosteatosis, but also myonecrosis. A prospective study using CT scans and muscle biopsies will have to further elucidate which changes in muscle are reflected by SMD in ICU patients.

A further limitation to our study is its observational design, precluding any deduction of causality. In addition, the complexity of critical illness may obscure residual confounding. Finally, the focus of our study was the prediction of long-term mortality at ICU admission, e.g. whether muscle quality at admission is a predictor of long-term mortality independent of muscle mass and of the best validated predictive score (APACHE). Further studies are needed to determine the risk factors for poor muscle quality and to determine the additional impact of ICU-acquired weakness on long-term mortality.

## Conclusions

Low skeletal muscle quality at ICU admission, as assessed by skeletal muscle density on CT scans, is associated with higher 6-month mortality in mechanically ventilated patients, independent of muscle quantity, APACHE II score, and BMI. Low muscle quality was also associated with longer hospital length of stay in survivors. Therefore, muscle quality appears to be as important for outcome as muscle quantity. Future intervention studies, including nutrition and early exercise, should not only focus on preventing further deterioration of muscle quantity, but also of muscle quality.

## Abbreviations

APACHE: Acute Physiological, Age, and Chronic Health Evaluation
BMI: Body mass index
CI: Confidence interval
CT: Computed tomography
Hospital-LOS: Hospital length of stay
HR: Hazard ratio
HU: Hounsfield Units
ICU: Intensive care unit
ICU-LOS: ICU length of stay
IMAT: Intermuscular adipose tissue
LOV: Length of ventilation
SMD: Skeletal muscle density
